# Estimating the Causal Effect of Tooth Loss on the Critical Outcome of COVID-19

**DOI:** 10.1177/23800844251353103

**Published:** 2025-07-17

**Authors:** N. Su, J.P.T.F. Ho, M. Ceylan, A.M.E. Schorer, H.C.M. Donders, T.L.T. Klausch, J.de Lange, B.G. Loos

**Affiliations:** 1 Department of Oral Public Health, Academic Center for Dentistry Amsterdam (ACTA), University of Amsterdam and Vrije Universiteit Amsterdam, Amsterdam, Noord-Holland, The Netherlands; 2 Department of Oral and Maxillofacial Surgery, Amsterdam UMC, Academic Centre for Dentistry Amsterdam (ACTA), University of Amsterdam and Vrije Universiteit Amsterdam, Amsterdam, The Netherlands; 3 Department of Oral and Maxillofacial Surgery, Northwest Clinics, Alkmaar, The Netherlands; 4 Department of Periodontology, Academic Center for Dentistry Amsterdam (ACTA), University of Amsterdam and Vrije Universiteit Amsterdam, Amsterdam, The Netherlands; 5 Department of Oral and Maxillofacial Surgery, Isala Zwolle, Zwolle, The Netherlands; 6 Department of Epidemiology and Data Science, Amsterdam UMC, Vrije Universiteit Amsterdam, Amsterdam, The Netherlands

**Keywords:** number of teeth, periodontitis, SARS-CoV-2, prognosis, propensity score

## Abstract

The objective of this study is to estimate the causal effect of tooth loss on the critical outcome of COVID-19, using 3 different propensity score methods. This retrospective study included patients aged >35 y with a diagnosis of COVID-19 between January 2020 and July 2021 at 2 hospitals in the Netherlands. The independent variable was number of teeth, dichotomized into 0 to 20 teeth (treatment) and 21 to 28 teeth (control). The critical outcome of COVID-19 (intensive care unit [ICU] admission and/or death) was the dependent variable. Potential confounders included patients’ demographics, lifestyle habits, medical conditions, COVID-19–related parameters, and hospitals. Three different propensity score methods were used to balance the baseline characteristics between the treatment and control groups: including propensity score matching (PSM), inverse propensity score weighting (IPW), and marginal mean weighting through stratification (MMWS). Both univariate and multivariate logistic regression analyses were performed to assess the causal association between tooth loss and the critical outcome of COVID-19 after the propensity methods. A total of 399 patients were included in the analyses. The multivariate logistic regression analysis controlling for the confounders revealed a statistically significant association between tooth loss and the critical outcome of COVID-19 across all the 3 propensity score methods: PSM (causal risk ratio [cRR]: 2.00; 95% confidence interval [CI]: 1.07–3.74; *P* = 0.03), MMWS (cRR: 1.78; 95% CI: 1.07–2.06; *P* = 0.03), and IPW (cRR: 1.85; 95% CI: 1.09–3.15; *P* = 0.02). Tooth loss has a statistically significant causal effect on the critical outcome of COVID-19. Patients with fewer teeth have a higher risk of ICU admission or mortality due to COVID-19.

**Knowledge Transfer Statement:**

The findings of this study can help clinicians and policymakers recognize the important role of oral health in COVID-19 prognosis. By encouraging health care professionals to integrate oral health assessments into comprehensive evaluations, the study promotes more accurate risk stratification for COVID-19 prognosis. This enables early interventions and better management of high-risk patients, ultimately leading to improved health outcomes by preventing critical outcomes of COVID-19 and enhancing patient care.

## Introduction

The novel coronavirus disease 2019 (COVID-19) pandemic has posed an important and urgent threat to global health since its outbreak in late 2019. As of April 27, 2025, COVID-19 has resulted in more than 777 million cumulative cases and 7.1 million deaths worldwide (WHO 2025). Although COVID-19 is no longer considered a global health emergency as of May 2023, it remains imperative to maintain vigilance and address its ongoing impacts and potential resurgence, particularly among high-risk groups. This is due to the potential for new variants, incomplete global vaccination coverage, and the need to monitor the long-term impacts of the pandemic (National Institute for Public Health and the Environment [RIVM] 2024; World Health Organization [WHO] 2024).

Tooth loss is considered a risk factor for respiratory diseases based on previous research. Chen et al. (2020) reported that every 5 increments in tooth loss was associated with 10% increased lung cancer risk based on a systematic review including 263,238 participants from 12 studies. In addition, Ciardo et al. (2024) showed a significant association between tooth loss and severe chronic obstructive pulmonary disease (COPD) based on a case-control study. Donders et al. (2022) also found a significant unadjusted association between tooth loss and severity of COVID-19. However, the association was absent when adjusting for the confounders, including age, gender, and cardiovascular disease. Tooth loss is also commonly used as a clinical indicator of oral health (Matsuyama et al. 2021; Beukers et al. 2021; Høvik et al. 2022), as the predominant causes of tooth loss in adults are oral inflammatory diseases, including periodontal diseases, caries, and periapical diseases (Broers et al. 2022). Preexisting, low-grade chronic inflammation has been highlighted as a key driver for severe COVID-19 outcomes (Baima et al. 2022). Multiple previous clinical studies have shown a significant association between these tooth loss–related oral inflammatory diseases and the critical outcome of COVID-19 (Baima et al. 2022; Costa et al. 2022; Moradi Haghgoo et al. 2023; Poyato-Borrego et al. 2023).

Despite the critical relevance of oral health for quality of life and well-being, the prevention and management of oral diseases have been severely neglected and marred by significant policy failures (Watt et al. 2019). One of the primary reasons for this neglect could be the previous lack of causal evidence linking oral health to other major relevant health outcomes (Matsuyama et al. 2021). Although earlier clinical studies have shown a statistical association between oral diseases and critical outcomes of COVID-19, no clinical studies to date have provided causal evidence for this association due to the observational nature of the research. However, understanding the possible causal links between oral diseases and COVID-19 is crucial for better targeting the prevention and treatment of these conditions and for advocating the prioritization of oral health on the global health policy agenda (Matsuyama et al. 2021).

Traditionally, causality cannot be determined in observational studies due to the confounding and lack of interchangeability among patients (Austin 2011). The propensity score analysis (PSA) attempts to reduce selection bias and to control for known confounding factors between treatment and control groups in observational studies. This can be achieved by balancing the observed baseline confounders among participants using propensity scores (Lalani et al. 2020). The PSA enables researchers to mimic some characteristics of a randomized controlled trial (RCT) in the context of an observational study, thereby strengthening causal inferences in observational studies by reducing selection bias (Austin 2011).

Therefore, the present study aims to estimate the causal effect of tooth loss on the critical outcome of COVID-19, based on 3 different propensity score methods, including propensity score matching (PSM), inverse propensity score weighting (IPW), and marginal mean weighting through stratification (MMWS). These methods allow for a robust analysis that controls for confounding factors, offering new insights into the role of oral health in COVID-19 severity.

## Methods

This study was approved by the Medical Ethics Committee of Isala Academy, Zwolle, the Netherlands (200710), on February 22, 2021, and taken over by Northwest Academy, Alkmaar, the Netherlands (L021-054), on September 20, 2021. The study followed the STROBE reporting guideline (von Elm et al. 2008).

### Study Design and Participants

The study was designed as a retrospective cohort study. We included consecutive hospitalized patients and outpatients from Isala hospital (Zwolle, the Netherlands) diagnosed with COVID-19 between January 2020 and May 2021, and from Northwest Clinics (Alkmaar, the Netherlands) diagnosed with COVID-19 between January 2020 and July 2021. The eligibility criteria were as follows:

- Patients were adults aged ≥35 y, as this age group is most likely to exbibit advanced periodontal disease with substantial tooth loss (Ramírez et al. 2017).
- Patients received an orthopantomogram (OPG) within 5 y prior to the COVID-19 diagnosis at the Department of Oral and Maxillofacial Surgery of the hospitals.
- Patients were diagnosed with COVID-19 based on a positive SARS-CoV-2 real-time reverse transcription-polymerase chain reaction test using swab material, sputum, or bronchoalveolar lavage samples.

### Outcome (Dependent Variable)

The dependent variable of the study was the presence or absence of the critical outcome of COVID-19. The course and outcomes of COVID-19 were classified into (1) ambulatory; (2) hospitalization without intensive care unit (ICU) admission; (3) ICU admission, and (4) death, based on the WHO Clinical Progression Scale (WHO Working Group on the Clinical Characterisation and Management of COVID-19 Infection 2020). The critical outcome was defined as ICU admission and/or death due to COVID-19, while the noncritical outcome was defined as ambulatory or hospitalization without ICU admission (Zhao et al. 2020; Su et al. 2023).

### Treatment (Independent Variable)

The independent variable was the number of remaining natural teeth, excluding third molars (ranging from 0 to 28). The number of present natural teeth was measured by counting all teeth visible on the OPGs including radices relicta. Pontics of fixed partial dentures and prosthetic dentures were not counted as teeth. The counting of the teeth was performed by the authors N.S., M.C., and B.L. In the study, the number of remaining teeth was dichotomized into 0 to 20 teeth (the “treatment” group) and 21 to 28 teeth (the “control” group), because 20 teeth is widely regarded as the cutoff for a functional dentition, and this cutoff is commonly used in clinical settings by nurses and triage doctors during patient admission (Kimble et al. 2022; Yan et al. 2022 Alobaidi et al. 2025).

### Confounders

The potential confounders included patients’ demographics, lifestyle habits, medical conditions, and COVID-19–related parameters. The confounders were determined based on prior knowledge and previous literature.

Patients demographics included age at the time of COVID-19 diagnosis, gender, socioeconomical status (SES) of the neighborhood where patients lived, and body mass index (BMI). SES was measured using normalized socioeconomical scores (−1 to +1) derived from the patients’ ZIP codes based on the Dutch Central Bureau of Statistics in 2019 (Centraal Bureau voor de Statistiek 2021). The scores were calculated based on financial prosperity, educational level, and recent employment history of private households. Higher scores indicated a higher SES. Lifestyle habits included smoking status based on the patients’ health records. Medical conditions included the presence or absence of diabetes mellitus, COPD, cardiovascular disease (CVD), obstructive sleep apnea, chronic kidney disease, hypertension, and hypercholesterolemia. Information on medical conditions was first collected from the electronic health records. If a medical condition was not mentioned in a patient file but the corresponding medication was documented (e.g., metformin and/or insulin, statins, and antihypertensive drugs), the patient was considered to have that medical condition. COVID-19–related parameters included vaccination status of the patients based on the health records and the dominant SARS-CoV-2 variants in the Netherlands at the time of COVID-19 diagnosis based on coronavirus dashboard of Dutch government (RIVM 2024). In addition, the hospital (Isala or Northwest Clinics) was considered a confounder.

### Statistical Analysis

The propensity score is defined as the probability of treatment assignment conditional on observed baseline confounders (Austin 2011). In this study, the propensity score represents the probability that a patient would have 0 to 20 teeth, conditional on the observed baseline confounders. A PSA aims to balance the distributions of the baseline confounders between the treatment and control groups by conditioning on the propensity score in the statistical analysis. This can reduce selection bias and known confounding between the groups, emulating randomized treatment assignment and enabling causal treatment effects estimation (Austin 2011; Lalani et al. 2020). Several propensity score methods are available; however, no consensus on a gold standard has been established (Schafer and Kang 2008). Therefore, this study used 3 different PSAs (PSM, IPW, and MMWS) to estimate the effect of the tooth loss on critical outcome of COVID-19. The choice for these methods aligns with previous studies (Austin 2011; Jongeneel et al. 2020) that demonstrated good performance in analyzing observational data (Austin 2011). The PSAs allow for unbiased estimations of causal treatment effects under the assumption of no unobserved confounding (Rosenbaum and Rubin 1983). Although this assumption cannot be formally tested, the most relevant confounders in the association between tooth loss and critical outcome of COVID-19, as reported in the literature, were available in the dataset. Detailed procedures for the PSAs are provided in the appendix. These include propensity score generation, overlap assessment of propensity scores between the groups, data trimming to improve overlap, balance assessment using standardized mean differences (SMD), additional regression adjustment for confounders, sensitivity analysis with E-values (VanderWeele and Ding 2017), and multiple imputation for missing data.

SPSS software 29 (IBM, New York, NY, USA) and R-Studio software 4.2.1 (RStudio Team, Boston, MA, USA) were used to perform the analyses.

## Results

### Characteristics of Included Patients

**Figure 1.**
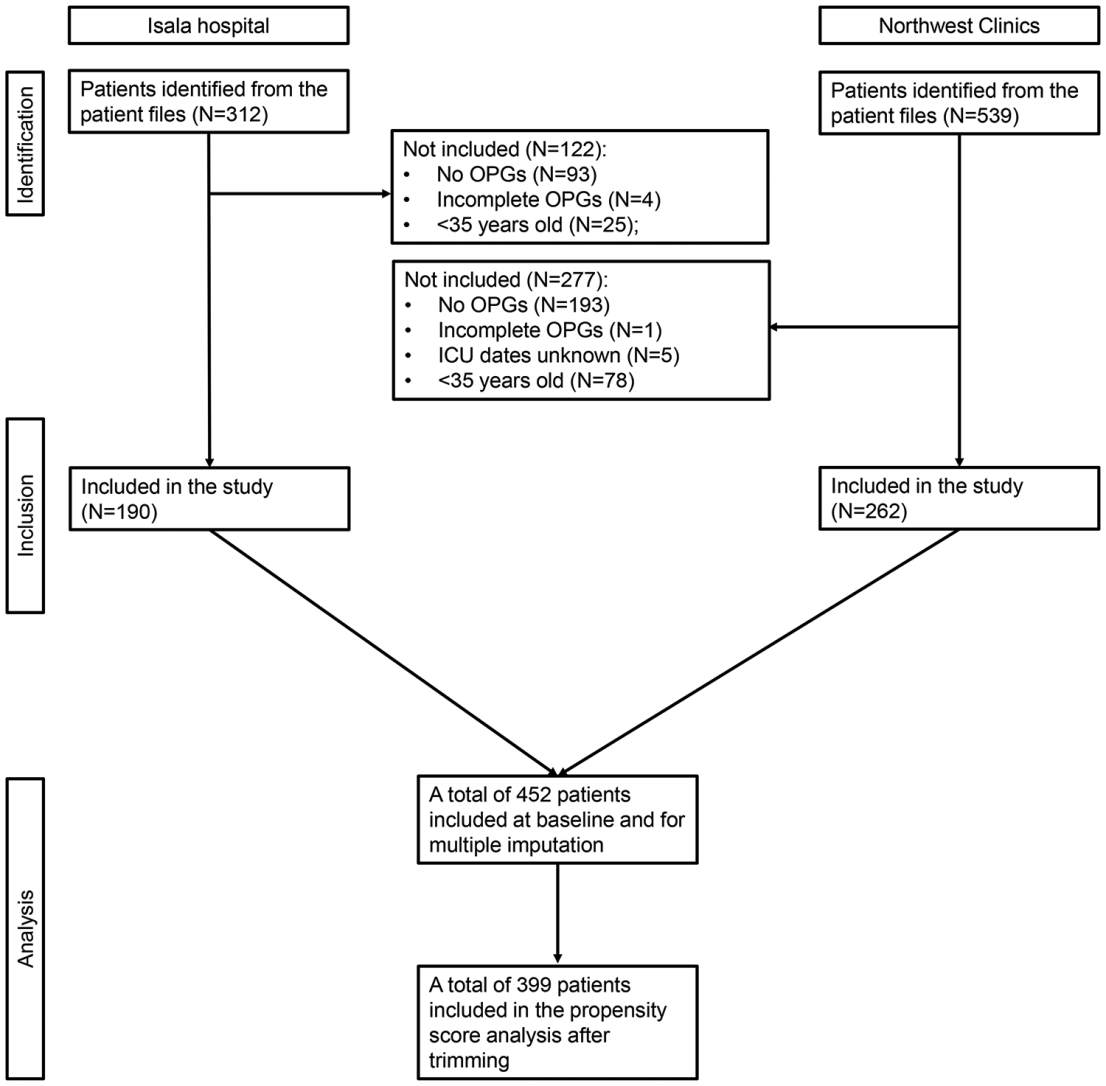
Flowchart of inclusion of the patients. *N* values represent the number of patients at each stage of the protocol.

A total of 452 patients were initially included for multiple imputation at baseline, while 399 patients were included in the PSAs after trimming. Consequently, 53 patients (11.7%) were excluded due to trimming. Figure 1 presents the flowchart of the inclusion of the patients.

Table 1 summarizes the main characteristics of the patients before trimming (the full dataset) and after trimming (the trimmed dataset). In the full dataset, there were 227 females (50.2%) and 225 males (49.8%), with a mean ± standard deviation (SD) age of 63.9 ± 14.8 y. In addition, 272 patients (60.2%) had >20 teeth, while 180 patients (39.8%) had <20 teeth. A total of 76 patients (16.8%) acquired critical outcome of COVID-19. In the trimmed dataset, there were 197 females (49.3%) and 202 males (50.7%), with a mean ± SD age of 64.4 ± 13.3 y. A total of 244 patients (61.1%) had >20 teeth, while 155 patients (38.9%) had <20 teeth. A total of 65 patients (16.3%) acquired a critical outcome of COVID-19. Overall, the main characteristics of the patients were similar between the full and trimmed datasets.

**Table 1.** Characteristics of the Independent Variable and Confounders Based on the Full Dataset (*N* = 452) and Trimmed Dataset (*n* = 399).

Variable	Full Dataset			Trimmed Dataset		
	Total (*N* = 452)	Outcome		Total (*n* = 399)	Outcome	
		Noncritical Outcome (*n* = 376)	Critical Outcome (*n* = 76)		Noncritical Outcome (*n* = 334)	Critical Outcome (*n* = 65)
Independent variable						
Number of remaining natural teeth						
>20 teeth	272 (60.2%)	251 (92.3%)	21 (7.7%)	244 (61.1%)	223 (91.4%)	21 (8.6%)
0–20 teeth	180 (39.8%)	125 (69.4%)	55 (30.6%)	155 (38.9%)	111 (71.8%)	44 (28.2%)
Confounders						
Age at diagnosis	63.9 (14.8)	61.6 (14.5)	75.1 (10.6)	64.4 (13.3)	62.7 (13.1)	73.1 (10.2)
Gender						
Female	227 (50.2%)	202 (89.0%)	25 (11.0%)	197 (49.3%)	175 (89.3%)	21 (10.7%)
Male	225 (49.8%)	174 (77.3%)	51 (22.7%)	202 (50.7%)	159 (78.4%)	44 (21.6%)
Socioeconomic status scores	0.049 (0.257)	0.057 (0.259)	0.012 (0.246)	0.056 (0.258)	0.060 (0.262)	0.036 (0.244)
BMI	27.3 (5.71)	27.2 (5.90)	27.6 (5.06)	27.4 (5.70)	27.3 (5.95)	27.8 (6.26)
Smoking						
Nonsmokers	256 (56.6%)	215 (84.0%)	41 (16.0%)	224 (56.1%)	189 (84.4%)	35 (15.6%)
Current smokers	29 (6.4%)	24 (82.8%)	5 (17.2%)	25 (6.3%)	20 (80.6%)	5 (19.4%)
Former smokers	167 (37.0%)	137 (82.0%)	30 (18.0%)	150 (37.6%)	125 (83.4%)	25 (16.6%)
Diabetes						
No	369 (81.6%)	322 (87.3%)	47 (12.7%)	326 (81.6%)	284 (87.3%)	42 (12.7%)
Yes	83 (18.4%)	54 (65.1%)	29 (34.9%)	73 (18.4%)	50 (68.3%)	23 (31.7%)
COPD						
No	367 (81.2%)	309 (84.2%)	58 (15.8%)	321 (80.5%)	271 (84.4%)	50 (15.6%)
Yes	85 (18.8%)	67 (78.8%)	18 (21.2%)	78 (19.5%)	63 (81.0%)	15 (19.0%)
CVD						
No	319 (70.6%)	280 (87.8%)	39 (12.2%)	284 (71.1%)	247 (87.3%)	36 (12.7%)
Yes	133 (29.4%)	96 (72.2%)	37 (27.8%)	115 (28.9%)	87 (75.1%)	29 (24.9%)
OSA						
No	397 (87.8%)	333 (83.9%)	64 (16.1%)	346 (86.6%)	293 (84.7%)	53 (15.3%)
Yes	55 (12.2%)	43 (78.2%)	12 (21.8%)	53 (13.4%)	41 (77.5%)	12 (22.5%)
Chronic kidney disease						
No	403 (89.2%)	344 (85.4%)	59 (14.6%)	357 (89.4%)	306 (85.8%)	51 (14.2%)
Yes	49 (10.8%)	32 (65.3%)	17 (34.7%)	42 (10.6%)	28 (66.7%)	14 (33.3%)
Hypertension						
No	256 (56.6%)	232 (90.6%)	24 (9.4%)	222 (55.6%)	201 (90.6%)	21 (9.4%)
Yes	196 (43.4%)	144 (73.5%)	52 (26.5%)	177 (44.4%)	133 (75.2%)	44 (24.8%)
Hypercholesterolemia						
No	357 (79.0%)	305 (85.4%)	52 (14.6%)	310(77.8%)	266 (85.7%)	44 (14.3%)
Yes	95 (21.0%)	71 (74.7%)	24 (25.3%)	89 (22.2%)	68 (77.1%)	21 (22.9%)
Vaccination						
Not vaccinated	329 (72.9%)	265 (80.5%)	64 (19.5%)	290 (72.7%)	234 (80.7%)	56 (19.3%)
Vaccinated	10 (2.2%)	7 (70.0%)	3 (30.0%)	9 (2.3%)	7 (77.3%)	2 (22.7%)
Unknown	113 (24.9%)	104 (92.0%)	9 (8.0%)	100 (25.0%)	93 (93.1%)	7 (6.9%)
Dominant variant						
Other	264 (58.4%)	208 (78.8%)	56 (21.2%)	231 (57.8%)	183 (79.4%)	48 (20.6%)
Alpha or Delta	188 (41.6%)	168 (89.4%)	20 (10.6%)	168 (42.2%)	151 (89.8%)	17 (10.2%)
Hospital						
Isala	190 (42.0%)	143 (75.3%)	47 (24.7%)	168 (42.1%)	131 (78.3%)	37 (21.7%)
Northwest Clinics	262 (58.0%)	233 (88.9%)	29 (11.1%)	231 (57.9%)	203 (87.7%)	28 (12.3%)

BMI, body mass index; COPD, chronic obstructive pulmonary disease; CVD, cardiovascular disease; OSA, obstructive sleep apnea.

### Overlap Assessment

**Figure 2.**
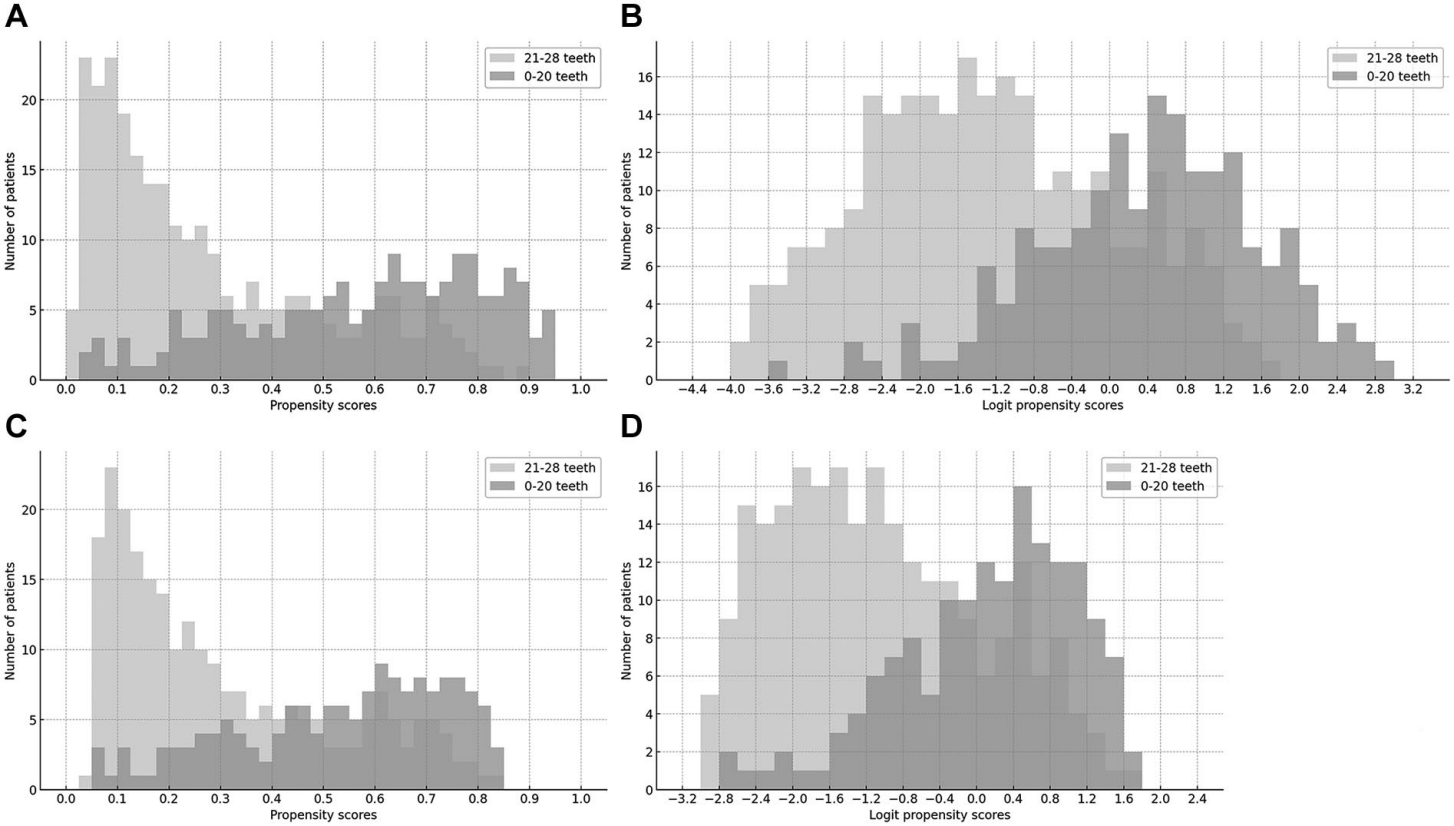
Overlap assessment between the treatment and control groups. (**A**) Overlap based on propensity scores before trimming. (**B**) Overlap based on logit propensity scores before trimming. (**C**) Overlap based on propensity scores after trimming. (**D**) Overlap based on logit propensity scores after trimming.

Figure 2 shows the overlap (common support) of the distributions of propensity scores and logit propensity scores between treatment and control groups, based on histograms. The overlap was poor before trimming (Figure 2A and B) but improved significantly after trimming, indicating good overlap in the distributions of both propensity scores and logit propensity scores after trimming (Figure 2C and D).

### Balance Assessment

Table 2 and Appendix Figure 1 presents the unadjusted and adjusted SMDs between the treatment and control groups for each confounder and logit propensity score (distance). The unadjusted SMDs between the 2 groups were large for most confounders and the logit propensity score (>0.1), indicating poor balance before propensity score analyses were conducted. However, after conducting PSM, MMWS, and IPW, the adjusted SMDs for all confounders and the logit propensity score were <0.10, indicating good balance between the 2 groups at baseline.

**Table 2.** Balance of Logit Propensity Scores and Confounders between 0–20 Teeth and 21–28 Teeth Based on PSM, IPW, and MMWS (*n* = 399).

	0–20 Teeth (*n* = 244)	>20 Teeth (*n* = 155)	Unadjusted Standardized Mean Difference	Adjusted Standardized Mean Difference		
				PSM	MMWS	IPW
Logit propensity scores (distance)	−1.0451 (1.1503)	0.1596 (1.0272)	1.1363 (0.7583^a^)	0.0031 (0.9918^a^)	0.0632 (1.1205^a^)	−0.0153 (1.0194^a^)
Age	59.97 (12.43)	71.27 (11.27)	0.9658 (0.8141^a^)	0.0077 (0.9386^a^)	0.0496 (1.0619^a^)	−0.0165 (0.9790^a^)
Gender						
Female	123 (50.4%)	74 (47.5%)				
Male	121 (49.6%)	81 (52.5%)	0.0295	−0.0565	0.0094	−0.0171
Socioeconomic status scores	0.0716 (0.2523)	0.0318 (0.2659)	−0.1549 (1.1241^a^)	−0.0624 (1.0440^a^)	−0.0326 (0.9761^a^)	−0.0211 (0.9283^a^)
BMI	27.43 (5.81)	27.38 (5.25)	−0.0119 (1.0181^a^)	−0.0898 (0.9826^a^)	0.0543 (1.1280^a^)	0.0632 (1.1414^a^)
Smoking						
Nonsmokers	150 (61.6%)	74 (47.4%)	−0.1412	0.0199	−0.0087	0.0121
Current smokers	16 (6.5%)	9 (6.0%)	−0.0047	−0.0032	−0.0127	−0.0103
Former smokers	78 (31.9%)	72 (46.5%)	0.1460	−0.0167	0.0214	−0.0018
Diabetes						
No	206 (84.4%)	120 (77.2%)				
Yes	38 (15.6%)	35 (22.8%)	0.0720	−0.0239	0.0280	0.0471
COPD						
No	203 (83.2%)	118 (76.3%)				
Yes	41 (16.8%)	37 (23.7%)	0.0690	0.0029	−0.0102	−0.0039
CVD						
No	190 (77.7%)	94 (60.7%)				
Yes	54 (22.3%)	61 (39.3%)	0.1698	−0.0088	0.0563	0.0159
OSA						
No	212 (87.1%)	133 (85.9%)				
Yes	32 (12.9%)	22 (14.1%)	0.0121	−0.0153	−0.0223	−0.0174
Chronic kidney disease						
No	220 (90.1%)	137 (88.3%)				
Yes	24 (9.9%)	18 (11.7%)	0.0179	0.0226	−0.0007	−0.0052
Hypertension						
No	154 (63.0%)	68 (44.0%)				
Yes	90 (37.0%)	87 (56.0%)	0.1899	−0.0309	0.0510	0.0205
Hypercholesterolemia						
No	200 (81.8%)	111 (71.5%)				
Yes	44 (18.2%)	44 (28.5%)	0.1034	−0.0203	0.0405	0.0357
Vaccination						
Not vaccinated	173 (71.0%)	117 (75.3%)	0.0434	−0.0482	0.0290	0.0113
Vaccinated	5 (2.1%)	4 (2.6%)	0.0056	0.0074	−0.0034	−0.0026
Unknown	66 (27.0%)	34 (22.1%)	-0.0490	0.0409	−0.0256	−0.0087
Dominant variant						
Other	134 (54.9%)	97 (62.5%)				
Alpha or Delta	110 (45.1%)	58 (37.5%)	−0.0760	0.0037	−0.0121	−0.0133
Hospital						
Isala	91 (37.5%)	76 (49.3%)				
Northwest Clinics	153 (62.5%)	79 (50.7%)	−0.1181	0.0162	0.0010	0.0084

BMI, body mass index; COPD, chronic obstructive pulmonary disease; CVD, cardiovascular disease; OSA, obstructive sleep apnea; PSM, propensity score matching; IPW, inverse propensity score weighting; MMWS, marginal mean weighting through stratification.

a Variance ratio.

### Causal Association between Tooth Loss and Critical Outcome of COVID-19

The logistic regression analysis without adjustment of confounders revealed no statistically significant association between tooth loss and the critical outcome of COVID-19 based on the 3 propensity score methods: PSM (causal risk ratio [cRR]: 1.84; 95% confidence interval [CI]: 0.85–3.98; *P* = 0.12), MMWS (cRR: 1.71; 95% CI: 1.00–2.92; *P* = 0.05), and IPW (cRR: 1.67; 95% CI: 0.97–2.99; *P* = 0.06) (Table 3).

**Table 3.** Causal Association between Tooth Loss and Critical Outcome of COVID-19 (*n* = 399).

	Without Regression Adjustment			With Regression Adjustment		
	cRR (95% CI)	*P* Value	E Value (Lower Limit of 95% CI)	cRR (95% CI)	*P* Value	E Value (Lower Limit of 95% CI)
PSM	1.84 (0.85–3.98)	0.12	3.09 (1.00)	2.00 (1.07–3.74)	0.03*	3.41 (1.34)
MMWS	1.71 (1.00–2.92)	0.05	2.80 (1.00)	1.78 (1.07–2.96)	0.03*	2.96 (1.34)
IPW	1.67 (0.97–2.88)	0.06	2.74 (1.00)	1.85 (1.09–3.15)	0.02*	3.11 (1.40)

CI, confidence interval; cRR, causal risk ratio; IPW, inverse propensity score weighting; MMWS, marginal mean weighting through stratification; PSM, propensity score matching.

* *P* < 0.05

The logistic regression analysis with adjustment of confounders revealed that tooth loss was statistically significantly associated with the critical outcome of COVID-19 based on all the 3 propensity score methods: PSM (cRR: 2.00; 95% CI: 1.07–3.74; *P* = 0.03), MMWS (cRR: 1.78; 95% CI: 1.07–2.06; *P* = 0.03), IPW (cRR: 1.85; 95% CI: 1.09–3.15; *P* = 0.02) (Table 3). This indicates that patients with 0 to 20 teeth had 1.78 to 2.00 times higher risks to acquire the critical outcome of COVID-19, compared with those with 21 to 28 teeth.

### Sensitivity Analysis for Unobserved Confounders

The E values based on the adjusted cRRs of PSM, MMWS, and IPW were 3.41, 2.96, and 3.11, respectively (Table 3). This indicates that the observed adjusted cRRs could be explained away by an unmeasured confounder that was associated with both tooth loss and critical outcome of COVID-19 by a RR of 2.96 to 3.41 fold each, above and beyond, but weaker confounding could not do so.

The lower limits of 95% CI of the E values were 1.34, 1.34, and 1.40, respectively (Table 3). This indicates that an unmeasured confounder associated with both tooth loss and critical outcome of COVID-19 by an RR of 1.34- to 1.40-fold each could explain away the lower confidence limit, but weaker confounding could not.

## Discussion

In the present study, we identified a significant causal effect of tooth loss on the critical outcome of COVID-19, using 3 different propensity score methods, including PSM, MMWS, and IPW. Patients with fewer teeth were found to have a significantly higher risk of ICU admission or mortality due to COVID-19. To the best of our knowledge, this is the first study to assess the potential causal association between tooth loss and critical outcome of COVID-19.

The number of teeth is a commonly used proxy variable for oral health in dental research. The leading reason for missing teeth is periodontal diseases (Broers et al. 2022). Based on the present study, tooth loss was considered a significant risk factor for severe COVID-19. An impaired masticatory function caused by tooth loss may increase the risk of poor nutritional status, particularly reduced calorie and protein intake (Kaurani et al. 2024), while long-term malnutrition may predispose patients to severe COVID-19 mainly through its integral role in immune function (Kurtz et al. 2021). In addition, to date, several biological mechanisms have been proposed in the etiopathogenetic link between tooth loss–related oral diseases (i.e., periodontal diseases) and the critical outcome of COVID-19. For instance, cytokine and microbial antigens released during periodontal infection contribute to the systemic proinflammatory state and may exacerbate COVID-19 prognosis (Romandini et al. 2018; Blanco-Melo et al. 2020; Villoria et al. 2024). Thus, if we consider patients with multiple teeth lost, they may still suffer from periodontitis at the remaining teeth in conjunction with a reduced chewing ability. It is interesting to note that SARS-CoV-2 has been detected in periodontal pockets, and these sites may act as reservoirs for the virus (Natto et al. 2022). Patients with periodontitis may have a higher viral load and could suffer from more severe COVID-19. In addition, periodontal disease can lead to an increased expression of angiotensin-converting enzyme 2 (ACE2) in the respiratory epithelium (Takahashi et al. 2020). Since ACE2 is a molecular target used by SARS-CoV-2 to infect human cells (Shang et al. 2020), this increased expression may enhance viral infectivity in the lower airways, potentially worsening the severity of COVID-19 (Takahashi et al. 2020; Baima et al. 2022).

The present study confirms the possible causal link between tooth loss and critical outcome of COVID-19, suggesting that tooth loss could be an important independent risk factor of vulnerability to severe COVID-19. This underscores the importance of maintaining good oral health, as well as early diagnosis and treatment of oral inflammatory diseases, especially periodontal diseases, in potentially reducing the risk of severe COVID-19. Moreover, these findings highlight the need for integrating oral health into broader medical assessments of COVID-19 risk and possibly during future pandemics in which systemic inflammation again plays a key role in disease progression. Given the possibility of COVID-19 coexisting with humans long-term, along with ongoing viral mutations and the uncertainty regarding the virulence of future variants, the findings of the present study are critically important for clinical practice.

### Strengths

In the present study, we employed propensity score methods, rather than traditional regression-based methods. Propensity score methods can reduce or eliminate the effects of confounding in observational studies and mimic an RCT to estimate causal treatment effects more accurately (Austin 2011). As compared with the regression-based methods, propensity score methods make it simple to assess whether the treatment and control groups have sufficient overlap in the distribution of baseline confounders (i.e., overlap assessment) and whether the groups are comparable by balancing confounders (i.e., balance assessment) (Austin 2011). Certain techniques can be used when overlap or balance is poor, which helps reduce confounding and improves the accuracy of causal effect estimates. In contrast, overlap and balance checks are difficult and not commonly performed in traditional regression-based methods (Austin 2011).

In the study, we used 3 different propensity score methods due to lack of consensus on a gold standard method (Schafer and Kang 2008). The cRRs based on the 3 methods were all statistically significant and similar in magnitude, ranging from 1.78 to 2.00. In addition, the E values, ranging from 2.96 to 3.41, provide reasonably strong evidence for causality, indicating that only moderate to substantial unmeasured confounders would reduce the observed significant association to null. This confirmed the robustness of the final results.

Another strength of this study is the use of number of teeth as a proxy for overall oral health. Number of teeth is an objective and easily measurable variable that is particularly useful in large-scale observational studies. By focusing on the number of teeth, this study highlights an easily measurable clinical marker that could be integrated into routine COVID-19 risk assessments in medical settings. We did not include specific oral diseases, such as periodontal diseases or caries, as the tested risk factors in the study, because diagnosing these diseases requires a professional dental examination by dentists, which could be challenging for medical clinicians.

### Limitations

Propensity score methods are typically designed to address confounding due to observed confounders. However, unobserved confounders may still exist between tooth loss and critical outcome of COVID-19, which were not accounted for in the analysis. For example, the oral conditions (e.g., periodontitis, periapical lesions, and caries), genetic variables, or serological biomarkers (e.g., C-reactive protein level, D-dimer, vitamin D3) (Takahashi et al. 2020; Gupta et al. 2022); were not included, which may bias the final results. Therefore, even if the word *causal* was used in the study, the causality needs to be further assessed and confirmed based on more comprehensive sets of confounders.

To improve the overlap between the treatment and control groups, 5% and 85% trimming was used to remove the patients with extreme propensity scores. Trimming is commonly used in propensity score methods (Lee et al. 2011) and can reduce bias by balancing the distribution of confounders between the 2 groups, leading to more reliable estimates of treatment effects and improving the internal validity of the analysis. However, trimming may lead to a narrower and less representative sample. The estimated treatment effect may not be generalized to the entire population but to only the subset of patients whose propensity scores lie within the trimmed range. In the present study, only a small percentage (11.7%) of the patients were excluded by trimming, and the main characteristics of the patients between the full and trimmed datasets were similar (Table 1). Therefore, trimming is not expected to substantially influence the representativeness of the remaining sample.

Future research is recommended to further investigate the association between the number of dental occluding pairs, the use of fixed prostheses, and critical outcomes of COVID-19, as this may more accurately reflect patients’ masticatory function and nutritional status.

In conclusion, this study provides the first causal evidence that tooth loss has a statistically significant impact on the critical outcome of COVID-19. Patients with 0 to 20 teeth may have a higher risk of ICU admission or mortality due to COVID-19 than those with 21 to 28 teeth do.

## Author Contributions

N. Su, contributed to conception, design, data acquisition, analysis, and interpretation, drafted the manuscript; J.P.T.F. Ho, contributed to conception, design, data acquisition, critically revised the manuscript; M. Ceylan, contributed to data acquisition and interpretation, critically revised the manuscript; A.M.E. Schorer, H.C.M. Donders, contributed to data acquisition, critically revised the manuscript; T.L.T. Klausch, contributed to design, data acquisition, analysis, and interpretation, critically revised the manuscript; J.de Lange, contributed to conception and design, critically revised the manuscript; B.G. Loos, contributed to conception, design, data acquisition and interpretation, critically revised the manuscript. All authors gave final approval and agree to be accountable for all aspects of the work.

## Supplemental Material

sj-docx-1-jct-10.1177_23800844251353103 – Supplemental material for Estimating the Causal Effect of Tooth Loss on the Critical Outcome of COVID-19 Supplemental material, sj-docx-1-jct-10.1177_23800844251353103 for Estimating the Causal Effect of Tooth Loss on the Critical Outcome of COVID-19 by N. Su, J.P.T.F. Ho, M. Ceylan, A.M.E. Schorer, H.C.M. Donders, T.L.T. Klausch, J. de Lange and B.G. Loos in JDR Clinical & Translational Research

## References

[bibr1-23800844251353103] Alobaidi F Heidari E Sabbah W. 2025. Health-related behaviour clusters and functional dentition in older people. Gerodontology [epub ahead of print 8 Jan 2025]. 10.1111/ger.12807. PMC12344633 39777740

[bibr2-23800844251353103] Austin PC . 2011. An introduction to propensity score methods for reducing the effects of confounding in observational studies. Multivariate Behav Res. 46(3):399–424. 21818162 10.1080/00273171.2011.568786 PMC3144483

[bibr3-23800844251353103] Baima G Marruganti C Sanz M Aimetti M Romandini M. 2022. Periodontitis and COVID-19: biological mechanisms and meta-analyses of epidemiological evidence. J Dent Res. 101(12):1430–1440. 35774019 10.1177/00220345221104725

[bibr4-23800844251353103] Beukers NGFM Su N Loos BG van der Heijden GJMG . 2021. Lower number of teeth is related to higher risks for ACVD and death—systematic review and meta-analyses of survival data. Front Cardiovasc Med. 8:621626. 10.3389/fcvm.2021.621626. 34026863 PMC8138430

[bibr5-23800844251353103] Blanco-Melo D Nilsson-Payant BE Liu WC Uhl S Hoagland D Møller R Jordan TX Oishi K Panis M Sachs D , et al. 2020. Imbalanced host response to SARS-COV-2 drives development of COVID-19. Cell. 181(5):1036–1045. 32416070 10.1016/j.cell.2020.04.026 PMC7227586

[bibr6-23800844251353103] Broers DLM Dubois L de Lange J Su N de Jongh A . 2022. Reasons for tooth removal in adults: a systematic review. Int Dent J. 72(1):52–57. 33648772 10.1016/j.identj.2021.01.011 PMC9275356

[bibr7-23800844251353103] Centraal Bureau voor de Statistiek (CBS). 2021. Sociaal-economische status; scores per wijk en buurt, regio-indeling 2021. The Hague (The Netherlands): CBS; [accessed 2024 March 24]. https://opendata.cbs.nl/#/CBS/nl/dataset/85163NED/table.

[bibr8-23800844251353103] Chen Y Zhu B Wu C Lin R Zhang X. 2020. Periodontal disease and tooth loss are associated with lung cancer risk. BioMed Res Int. 2020:5107696. 10.1155/2020/5107696. 32802852 PMC7403933

[bibr9-23800844251353103] Ciardo A Simon MM Eberhardt R Brock JM Ritz A Kim TS . 2024. Severe chronic obstructive pulmonary disease is associated with reduced oral health conditions. Oral Dis. 30(5):3400–3412. 37794640 10.1111/odi.14755

[bibr10-23800844251353103] Costa CA Vilela ACS Oliveira SA Gomes TD Andrade AAC Leles CR Costa NL . 2022. Poor oral health status and adverse COVID-19 outcomes: a preliminary study in hospitalized patients. J Periodontol. 93(12):1889–1901. 35294780 10.1002/JPER.21-0624 PMC9088593

[bibr11-23800844251353103] Donders HCM van der Sleen JM Kleinbergen YJ Su N de Lange J Loos BG . 2022. Alveolar bone loss and tooth loss are associated with COVID-19 severity but are not independent risk factors. An explorative study. Adv Oral Maxillofac Surg. 5:100223. 10.1016/j.adoms.2021.100223.

[bibr12-23800844251353103] Gupta S Mohindra R Singla M Khera S Sahni V Kanta P Soni RK Kumar A Gauba K Goyal K , et al. 2022. The clinical association between periodontitis and COVID-19. Clin Oral Investig. 26(2):1361–1374. 10.1007/s00784-021-04111-3 PMC8390180 34448073

[bibr13-23800844251353103] Høvik H Kolberg M Gjøra L Nymoen LC Skudutyte-Rysstad R Hove LH Sun YQ Fagerhaug TN . 2022. The validity of self-reported number of teeth and edentulousness among Norwegian older adults, the hunt study. BMC Oral Health. 22(1):82. 10.1186/s12903-022-02116-2. 35313882 PMC8935783

[bibr14-23800844251353103] Jongeneel G Klausch T van Erning FN Vink GR Koopman M Punt CJA Greuter MJE Coupé VMH . 2020. Estimating adjuvant treatment effects in stage II colon cancer: comparing the synthesis of randomized clinical trial data to real-world data. Int J Cancer. 146(11):2968–2978. 31424568 10.1002/ijc.32629 PMC7187209

[bibr15-23800844251353103] Kaurani P Kakodkar P Bhowmick A Samra RK Bansal V. 2024. Association of tooth loss and nutritional status in adults: an overview of systematic reviews. BMC Oral Health. 24(1):838. 10.1186/s12903-024-04602-1. 39049002 PMC11267674

[bibr16-23800844251353103] Kimble R McLellan G Lennon LT Papacosta AO Weyant RJ Kapila Y Mathers JC Wannamathee SG Whincup PH Ramsay SE . 2022. Association between oral health markers and decline in muscle strength and physical performance in later life: longitudinal analyses of two prospective cohorts from the UK and the USA. Lancet Healthy Longev. 3(11):e777–e788. 10.1016/s2666-7568(22)00222-7. PMC10397540 36356627

[bibr17-23800844251353103] Kurtz A Grant K Marano R Arrieta A Grant K Jr Feaster W Steele C Ehwerhemuepha L. 2021. Long-term effects of malnutrition on severity of COVID-19. Sci Rep. 11(1):14974. 10.1038/s41598-021-94138-z. 34294743 PMC8298504

[bibr18-23800844251353103] Lalani N Jimenez RB Yeap B. 2020. Understanding propensity score analyses. Int J Radiat Oncol Biol Phys. 107(3):404–407. 32531385 10.1016/j.ijrobp.2020.02.638

[bibr19-23800844251353103] Lee BK Lessler J Stuart EA . 2011. Weight trimming and propensity score weighting. PLoS One. 6(3):e18174. 10.1371/journal.pone.0018174. PMC3069059 21483818

[bibr20-23800844251353103] Matsuyama Y Jürges H Dewey M Listl S. 2021. Causal effect of tooth loss on depression: evidence from a population-wide natural experiment in the USA. Epidemiol Psychiatr Sci. 30:e38. 10.1017/s2045796021000287. PMC8157508 34030762

[bibr21-23800844251353103] Moradi Haghgoo J Torkzaban P Farhadian M Moosavi Sedeh SA . 2023. Association between the severity of periodontitis, COVID-19, C-reactive protein and interleukin-6 levels in hospitalized patients: a case–control study. BMC Oral Health. 23(1):556. 10.1186/s12903-023-03270-x. 37568161 PMC10422752

[bibr22-23800844251353103] National Institute for Public Health and the Environment (RIVM). 2024. Variants of the coronavirus SARS-CoV-2. Utrecht (The Netherlands): RIVM; [updated 2025 May 28; accessed 2024 March 21]. http://www.rivm.nl/en/coronavirus-covid-19/current/variants.

[bibr23-23800844251353103] Natto ZS Afeef M Bakhrebah MA Ashi H Alzahrani KA Alhetheel AF Fletcher HM . 2022. Can periodontal pockets and caries lesions act as reservoirs for coronavirus? Mol Oral Microbiol. 37(2):77–80. 35060684 10.1111/omi.12362 PMC9237656

[bibr24-23800844251353103] Poyato-Borrego M León-López M Martín-González J Cisneros-Herreros JM Cabanillas-Balsera D Segura-Egea JJ . 2023. Endodontic variables in patients with SARS-COV-2 infection (COVID-19) in relation to the severity of the disease. Med Oral Patol Oral Cir Bucal. 28(4):e355–e361. 10.4317/medoral.25773. PMC10314353 36641741

[bibr25-23800844251353103] Ramírez JC Lopera NS López AP Agudelo-Suárez AA Botero JE. 2017. Periodontal disease and its relationship with clinical and sociodemographic variables in adult patients treated in a service/teaching institution. Rev Odontol Mex. 21(3):165–172. http://dx.doi.org/10.1016/j.rodmex.2017.09.012.

[bibr26-23800844251353103] Romandini M Laforí A Romandini P Baima G Cordaro M. 2018. Periodontitis and platelet count: a new potential link with cardiovascular and other systemic inflammatory diseases. J Clin Periodontol. 45(11):1299–1310. 30133784 10.1111/jcpe.13004

[bibr27-23800844251353103] Rosenbaum PR Rubin DB . 1983. The central role of the propensity score in observational studies for causal effects. Biometrika. 70(1):41–55.

[bibr28-23800844251353103] Schafer JL Kang J. 2008. Average causal effects from nonrandomized studies: a practical guide and simulated example. Psychol Methods. 13(4):279–313. 19071996 10.1037/a0014268

[bibr29-23800844251353103] Shang J Wan Y Luo C Ye G Geng Q Auerbach A Li F. 2020. Cell entry mechanisms of SARS-COV-2. Proc Natl Acad Sci U S A. 117(21):11727–11734. 32376634 10.1073/pnas.2003138117 PMC7260975

[bibr30-23800844251353103] Su N Donders MHCM Ho JTF Vespasiano V de Lange J Loos BG . 2023. Development and external validation of prediction models for critical outcomes of unvaccinated COVID-19 patients based on demographics, medical conditions and dental status. Heliyon. 9(4):e15283. 10.1016/j.heliyon.2023.e15283. PMC10084632 37064437

[bibr31-23800844251353103] Takahashi Y Watanabe N Kamio N Kobayashi R Iinuma T Imai K. 2020. Aspiration of periodontopathic bacteria due to poor oral hygiene potentially contributes to the aggravation of COVID-19. J Oral Sci. 63(1):1–3. 33177276 10.2334/josnusd.20-0388

[bibr32-23800844251353103] VanderWeele TJ Ding P. 2017. Sensitivity analysis in observational research: introducing the E-value. Ann Intern Med. 167(4):268–274. 28693043 10.7326/M16-2607

[bibr33-23800844251353103] Villoria GE Fischer RG Tinoco EM Meyle J Loos BG. 2024. Periodontal disease: a systemic condition. Periodontol 2000. 96(1):7–19. 39494478 10.1111/prd.12616 PMC11579822

[bibr34-23800844251353103] von Elm E Altman DG Egger M Pocock SJ Gøtzsche PC Vandenbroucke JP STROBE Initiative . 2008. The Strengthening the Reporting of Observational Studies in Epidemiology (STROBE) statement: guidelines for reporting observational studies. J Clin Epidemiol. 61(4):344–349. 18313558 10.1016/j.jclinepi.2007.11.008

[bibr35-23800844251353103] Watt RG Daly B Allison P Macpherson LMD Venturelli R Listl S Weyant RJ Mathur MR Guarnizo-Herreño CC Celeste RK , et al. 2019. Ending the neglect of global oral health: time for radical action. Lancet. 394(10194):261–272. 31327370 10.1016/S0140-6736(19)31133-X

[bibr36-23800844251353103] World Health Organization. 2024. COVID-19 vaccination, world data. Geneva (Switzerland): WHO; [accessed 2024 March 21]. https://data.who.int/dashboards/covid19/vaccines.

[bibr37-23800844251353103] World Health Organization. 2025. WHO COVID-19 dashboard. Geneva (Switzerland): WHO; [accessed 2025 May 16]. https://data.who.int/dashboards/covid19/cases?n=c.

[bibr38-23800844251353103] World Health Organization Working Group on the Clinical Characterisation and Management of COVID-19 infection. 2020. A minimal common outcome measure set for COVID-19 clinical research. Lancet Infect Dis. 20(8):e192–e197. 10.1016/s1473-3099(20)30483-7. PMC7292605 32539990

[bibr39-23800844251353103] Yan GLK Tan MN Wong ML Tay CM Allen PF . 2022. Functional dentition, chronic periodontal disease and frailty in older adults-a narrative review. Int J Environ Res Public Health. 20(1):502. 10.3390/ijerph20010502. 36612820 PMC9819030

[bibr40-23800844251353103] Zhao Z Chen A Hou W Graham JM Li H Richman PS Thode HC Singer AJ Duong TQ . 2020. Prediction model and risk scores of ICU admission and mortality in COVID-19. PLoS One. 15(7):e0236618. 10.1371/journal.pone.0236618. PMC7392248 32730358

