# Supplementary material for: Estimating the Causal Effect of Tooth Loss on the Critical Outcome of COVID-19
Source: JDR Clin Trans Res. 2025 Jul 17;11(2):226–36. doi: 10.1177/23800844251353103 (PMC12967384; doi:10.1177/23800844251353103)
Supplement: sj-docx-1-jct-10.1177_23800844251353103 – Supplemental material for Estimating the Causal Effect of Tooth Loss on the Critical Outcome of COVID-19 [file sj-docx-1-jct-10.1177_23800844251353103.docx]

**Supplementary File**

**Estimating causal effect of tooth loss on critical outcome of COVID-19**

N. Su, J.P.T.F. Ho, M. Ceylan, A.M.E. Schorer, H.C.M. Donders, T.L.T. Klausch, J. de Lange, B.G. Loos

**Overlap, propensity score analyses, and balance**

Propensity scores were generated using a logistic regression model, where the dependent variable was the dichotomized number of teeth and the independent variables were all the confounders. We included only the main effects of the confounders in the model.

To assess the overlap between the treatment (i.e. 0-20 teeth) and control (i.e. 21-28 teeth) groups, we examined the distribution of propensity scores and logit propensity scores using histograms for both groups. Visual inspection was used to identify regions where the propensity scores of the treatment and control groups overlapped (common support). Due to unsatisfactory overlap in the original full dataset, we conducted trimming by removing individuals with extreme propensity scores, falling in the lower 5% and upper 85% of the distribution. Trimming enhances the comparability between the treatment and control groups by ensuring better overlap. Propensity scores were re-estimated based on the trimmed dataset. Three different PSAs (propensity score matching [PSM], inverse propensity score weighting [IPW], and marginal mean weighting through stratification [MMWS]) were then separately conducted on the trimmed dataset.

PSM is a method that creates matched sets of patients in the treatment group and the control group who share similar values of propensity scores (Rosenbaum and Rubin 1985). We used optimal full matching (OFM), a specific type of PSM. OFM is a form of sub-classification wherein all subjects in both treatment and control groups (i.e. the full samples), are assigned to a subclass and receive at least one match (Austin and Stuart 2015). The mean difference in propensity scores between groups is minimized in the matched set (Austin and Stuart 2015). The advantage of OFM over other conventional matching methods is that it does not reduce number of patients and it can provide superior performance (Austin and Stuart 2015).

IPW is a method that uses the propensity score to balance the baseline confounders between the treatment and control groups by weighting each patient with the inverse probability of receiving the actual treatment (i.e. 0-20 teeth) (Austin 2011). The propensity score weights were defined as w(x) = 1/p(x) for a treated patient and as w(x) = 1/(1-p(x)) for a control patient, where w(x) is the weight for each patient and p(x) is the propensity score (Austin 2011; Rosenbaum 1987).

The MMWS method is a newly non-parametric strategy that combines key elements of propensity score stratification and IPW (Hong 2010). In the method, patients are first stratified into four mutually exclusive subclasses based on the individual propensity scores. The weights of each stratum were calculated for treated and untreated patients separately. The weight for treated patients is the inverse probability of being in the treated group within the stratum, calculated as w = 1/p_s_, where p_s_ is the proportion of patients in the treated group in stratum s. The weight for untreated patients is the inverse probability of being in the untreated group in the stratum, calculated as w = 1/(1- p_s_).

As part of the PSA, we assessed covariate balance using standardized mean differences (SMDs) for each confounder and logit propensity scores (adjusted SMDs). The logit propensity score was used for the analysis because the logit of propensity score is more likely to be normally distributed than the propensity score itself, which can reduce the bias achieved by matching (Austin 2011; Rosenbaum and Rubin 1985). A confounder (or logit propensity score) was considered well-balanced if the absolute value of SMD was <0.1. If the SMDs of all the confounders and logit propensity score were <0.1, it indicated that the propensity score model has adequately balanced the confounders between the groups, similar to what one would expect after randomization in an RCT.

**Additional regression adjustment**

To assess the association between tooth loss and critical outcome of COVID-19 after matching, weighting, and stratification, logistic regression analysis without adjustment of the confounders was first performed. Afterward, we additionally used covariate-based regression-adjustment, which can confer the double-robustness property. This property provides protection against model misspecification and yields more precise estimates (Rosenbaum and Rubin 1984; Bang and Robins 2005). Specifically, logistic regression analyses were performed to assess the treatment effect by adjusting for the confounders. The unadjusted and adjusted causal risk ratios (cRRs) of tooth loss on the critical outcome of COVID-19 were calculated. Robust standard errors and 95% confidence intervals of the cRRs were generated to correct for the uncertainty in the weights. We estimated the average treatment effect (ATE) for the whole population (i.e. marginal cRRs), rather than the average treatment effect on the treated (ATT), using the R package *marginaleffects*.

**Sensitivity analysis**

As a sensitivity analysis, the E values were calculated for each propensity score method to estimate the robustness of the treatment effect against the influence of unobserved confounders (van der Weele and Ding 2017). The E value represents the minimum strength of association, on the risk ratio or odds ratio scale, that an unmeasured confounder would need to have with both the treatment and outcome to fully explain away a specific treatment-outcome association, conditional on the measured confounders (van der Weele and Ding 2017). A larger E value indicates a more robust observed treatment effect.

**Missing data**

The multiple imputation technique was used to address the missing data. To impute missing values, 35 imputed datasets with 10 iterations each were created, using predictive mean matching (PMM). The imputation model included all the abovementioned variables, including the dependent variable, the independent variable, and the confounders. In the imputation model, the original number of teeth and COVID-19 severity, rather than the dichotomized variables, were used. The final results from each imputed dataset were pooled based on Rubin`s rule.

Multiple imputation and descriptive statistics were conducted via SPSS software 29 (IBM, New York, USA). The propensity score analyses, including PSM, IPW, and MMWS, were all performed using R-Studio software 4.2.1 (RStudio Team, Boston, USA).

**References**

Austin PC. 2011. An introduction to propensity score methods for reducing the effects of confounding in observational studies. Multivariate Behav Res. 46(3):399-424.

Austin PC, Stuart EA. 2015. Optimal full matching for survival outcomes: A method that merits more widespread use. Stat Med. 34(30):3949–3967.

Bang H, Robins JM. 2005. Doubly robust estimation in missing data and causal inference models. Biometrics. 61(4):962–973.

Hong G. 2010. Marginal mean weighting through stratification: Adjustment for selection bias in Multilevel Data. J Educ Behav Stat. 35(5):499–531.

Rosenbaum PR. 1987. Model-based direct adjustment. J Am Stat Assoc. 82(398):387–394.

Rosenbaum PR, Rubin DB. 1984. Reducing bias in observational studies using subclassification on the propensity score. J Am Stat Assoc. 79(387):516–524.

Rosenbaum PR, Rubin DB. 1985. Constructing a control group using multivariate matched sampling methods that incorporate the propensity score. American Statistician. 39(1):33–38.

VanderWeele TJ, Ding P. 2017. Sensitivity analysis in observational research: Introducing the E-value. Ann Intern Med. 167(4):268-274.

**Appendix Figure 1**


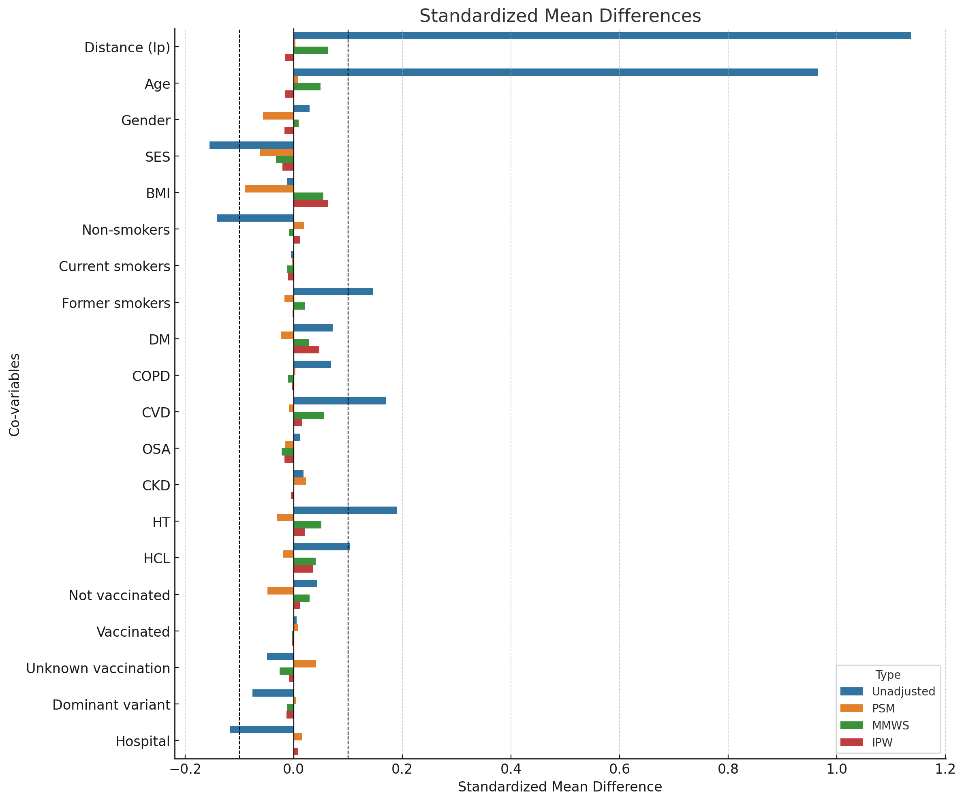


**Appendix Figure 1** The balance assessment between treatment and control groups for the dataset before using propensity score methods (unadjusted) and after using the three propensity score methods.

STROBE Statement—checklist of items that should be included in reports of observational studies

|  | **Item No** | **Recommendation** | **PAGE** |
| --- | --- | --- | --- |
| **Title and abstract** | 1 | (*a*) Indicate the study’s design with a commonly used term in the title or the abstract | NA |
|  |  | (*b*) Provide in the abstract an informative and balanced summary of what was done and what was found | 2 |
| **Introduction** | | |  |
| Background/rationale | 2 | Explain the scientific background and rationale for the investigation being reported | 3-4 |
| Objectives | 3 | State specific objectives, including any prespecified hypotheses | 4 |
| **Methods** | | |  |
| Study design | 4 | Present key elements of study design early in the paper | 4 |
| Setting | 5 | Describe the setting, locations, and relevant dates, including periods of recruitment, exposure, follow-up, and data collection | 4 |
| Participants | 6 | (*a*) *Cohort study*—Give the eligibility criteria, and the sources and methods of selection of participants. Describe methods of follow-up  *Case-control study*—Give the eligibility criteria, and the sources and methods of case ascertainment and control selection. Give the rationale for the choice of cases and controls  *Cross-sectional study*—Give the eligibility criteria, and the sources and methods of selection of participants | 4-5 |
|  |  | (*b*) *Cohort study*—For matched studies, give matching criteria and number of exposed and unexposed  *Case-control study*—For matched studies, give matching criteria and the number of controls per case | Supplementary file 1 |
| Variables | 7 | Clearly define all outcomes, exposures, predictors, potential confounders, and effect modifiers. Give diagnostic criteria, if applicable | 5-6 |
| Data sources/ measurement | 8* | For each variable of interest, give sources of data and details of methods of assessment (measurement). Describe comparability of assessment methods if there is more than one group | *5-6* |
| Bias | 9 | Describe any efforts to address potential sources of bias | Supplementary file 1 |
| Study size | 10 | Explain how the study size was arrived at | Not reported |
| Quantitative variables | 11 | Explain how quantitative variables were handled in the analyses. If applicable, describe which groupings were chosen and why | 5-6 |
| Statistical methods | 12 | (*a*) Describe all statistical methods, including those used to control for confounding | 6, supplementary file 1 |
|  |  | (*b*) Describe any methods used to examine subgroups and interactions | NA |
|  |  | (*c*) Explain how missing data were addressed | Supplementary file 1 |
|  |  | (*d*) *Cohort study*—If applicable, explain how loss to follow-up was addressed  *Case-control study*—If applicable, explain how matching of cases and controls was addressed  *Cross-sectional study*—If applicable, describe analytical methods taking account of sampling strategy | NA |
|  |  | (*e*) Describe any sensitivity analyses | Supplementary file 1 |

| **Results** | | | **Page** |
| --- | --- | --- | --- |
| Participants | 13* | (a) Report numbers of individuals at each stage of study—eg numbers potentially eligible, examined for eligibility, confirmed eligible, included in the study, completing follow-up, and analysed | Figure 1 |
|  |  | (b) Give reasons for non-participation at each stage | Figure 1 |
|  |  | (c) Consider use of a flow diagram | Figure 1 |
| Descriptive data | 14* | (a) Give characteristics of study participants (eg demographic, clinical, social) and information on exposures and potential confounders | Table 1 |
|  |  | (b) Indicate number of participants with missing data for each variable of interest | Table 1 |
|  |  | (c) *Cohort study*—Summarise follow-up time (eg, average and total amount) | NA |
| Outcome data | 15* | *Cohort study*—Report numbers of outcome events or summary measures over time | Table 1 |
|  |  | *Case-control study—*Report numbers in each exposure category, or summary measures of exposure | NA |
|  |  | *Cross-sectional study—*Report numbers of outcome events or summary measures | *NA* |
| Main results | 16 | (*a*) Give unadjusted estimates and, if applicable, confounder-adjusted estimates and their precision (eg, 95% confidence interval). Make clear which confounders were adjusted for and why they were included | 7-9 |
|  |  | (*b*) Report category boundaries when continuous variables were categorized | 4-5 |
|  |  | (*c*) If relevant, consider translating estimates of relative risk into absolute risk for a meaningful time period | NA |
| Other analyses | 17 | Report other analyses done—eg analyses of subgroups and interactions, and sensitivity analyses | 9 |
| **Discussion** | | |  |
| Key results | 18 | Summarise key results with reference to study objectives | 9 |
| Limitations | 19 | Discuss limitations of the study, taking into account sources of potential bias or imprecision. Discuss both direction and magnitude of any potential bias | 11-12 |
| Interpretation | 20 | Give a cautious overall interpretation of results considering objectives, limitations, multiplicity of analyses, results from similar studies, and other relevant evidence | 10-11 |
| Generalisability | 21 | Discuss the generalisability (external validity) of the study results | 11-12 |
| **Other information** | | |  |
| Funding | 22 | Give the source of funding and the role of the funders for the present study and, if applicable, for the original study on which the present article is based | 12 |

*Give information separately for cases and controls in case-control studies and, if applicable, for exposed and unexposed groups in cohort and cross-sectional studies.

**Note:** An Explanation and Elaboration article discusses each checklist item and gives methodological background and published examples of transparent reporting. The STROBE checklist is best used in conjunction with this article (freely available on the Web sites of PLoS Medicine at http://www.plosmedicine.org/, Annals of Internal Medicine at http://www.annals.org/, and Epidemiology at http://www.epidem.com/). Information on the STROBE Initiative is available at www.strobe-statement.org.
